# The Impact of Curing Conditions on the Microstructure and Resultant Macro-Performance of Manufactured Sand Concrete

**DOI:** 10.3390/ma19132698

**Published:** 2026-06-23

**Authors:** Hongmei Chen, Juan Zhou, Ronggui Liu, Jialei Wang, Yu Xiang

**Affiliations:** 1 School of Civil Engineering, Nantong Institute of Technology, Nantong 226000, China; 20210002@ntit.edu.cn (H.C.); wangjl@nit.zju.edu.cn (J.W.); 2 School of Business, Nantong Institute of Technology, Nantong 226000, China; 3 School of Traffic and Transportation Engineering, Xinjiang University, Urumqi 830017, China; 4 National Engineering Research Center of Highway Maintenance Technology, Changsha University of Science & Technology, Changsha 410114, China

**Keywords:** manufactured sand, concrete, steam curing, macroscopic performance, deformation, microstructure

## Abstract

This study comprehensively evaluates the mechanical properties, shrinkage behavior, and durability of concrete prepared with limestone- and granite-manufactured sands under standard-curing and steam-curing conditions. The results indicate that limestone-manufactured sand concrete consistently exhibits superior compressive strength and splitting tensile strength across all curing ages, outperforming granite-modified counterparts. The introduction of granite-manufactured sand significantly degrades these mechanical properties, with deterioration intensifying as granite content increases. Dynamic elastic modulus and damping ratio analyses reveal that limestone-based concrete maintains the highest dynamic stiffness and lowest energy dissipation under both curing regimes, suggesting fewer internal defects. In contrast, granite incorporation reduces the dynamic elastic modulus and increases the damping ratio, reflecting structural deterioration and enhanced energy loss. Drying shrinkage tests demonstrate that limestone concrete achieves the lowest shrinkage deformation throughout the testing period, even under steam-curing conditions. Conversely, granite addition markedly elevates shrinkage, particularly under steam-curing conditions, leading to compromised volumetric stability. Durability assessments highlight that manufactured sand concrete exhibits higher capillary absorption, electrical flux, and porosity, attributed to inherent material defects and the surface characteristics of manufactured sand. Granite-modified concrete further weakens interfacial shear strength between aggregates and cement paste, indicating poor interfacial bonding. Steam curing exacerbates microstructural defects, emphasizing the need to optimize curing protocols. The findings propose strategies for enhancing manufactured sand concrete performance: improving interfacial adhesion between aggregates and cement paste, rationalizing supplementary material dosages, and refining steam curing regimes. These measures offer potential pathways to develop high-performance manufactured sand concrete with balanced mechanical and durability properties.

## 1. Introduction

The investigation and application of manufactured sand concrete constitute a crucial pathway for alleviating resource scarcity, mitigating environmental pollution, and reducing carbon emissions in the construction industry. As an artificial substitute for conventional natural river sand, manufactured sand plays an increasingly vital role in concrete production. In the precast concrete industry, steam curing is universally adopted as a standard-curing regime to accelerate strength development and enhance production efficiency by shortening the mold turnover cycle. However, the mechanical crushing process used in its production from parent rocks results in manufactured sand particles exhibiting an angular morphology and rough surface texture, contrasting with the rounded morphology of water-eroded natural river sand. These inherent characteristics lead to significant challenges in concrete workability [1]. Furthermore, the diversity and complexity of raw material sources for manufactured sand contribute to substantial performance variations in resulting concrete mixtures. Despite the extensive research on manufactured sand concrete, there is a notable scarcity of systematic comparative studies focusing on the microstructural evolution—specifically regarding the interfacial transition zone (ITZ) and pore structure—of manufactured sand derived from distinct lithologies, such as limestone versus granite, under steam-curing conditions. Consequently, the performance characteristics of manufactured sand concrete under different curing conditions remain subject to considerable uncertainties. Current research by domestic and international scholars primarily focuses on three aspects: the workability, mechanical properties, and durability performance of manufactured sand concrete.

Manufactured sand concrete exhibits inherent challenges in workability, including poor cohesion, significant slump loss, and inconsistent fresh mixture performance. These issues primarily correlate with characteristic parameters of manufactured sand: particle morphology, gradation, powder content, clay content, and flocculant residues [2]. Notably, residual flocculants (e.g., polyacrylamide and polyaluminum chloride) in water-washed manufactured sand exacerbate slump loss over time and necessitate an increased superplasticizer dosage [3]. Scholars have implemented multiple mitigation strategies. (i) Supplementary cementitious materials: Incorporation of fly ash, slag, and silica fume with an optimized powder content significantly enhances the workability of C80 manufactured sand concrete while improving particle coating and anti-segregation properties [4,5,6,7,8]. (ii) Chemical composition-based classification: Zhuang et al. proposed categorizing manufactured sands by dominant oxide content (high-calcium, high-silicon, high-aluminum, and high-iron), enabling tailored admixture formulations to address specific workability deficiencies [9]. (iii) Mix proportion optimization: Through adjustments in the sand ratio and water-to-cement ratio (W/C), Zhu et al. achieved satisfactory workability in tunnel muck-based manufactured sand concrete [10]. (iv) Rheology modifiers: Introduction of viscosity-modifying agents effectively resolves bleeding, segregation, and poor pumpability in concrete prepared with wet-process manufactured sand [11]. These approaches collectively demonstrate that systematic optimization of material composition and production processes can counteract the inherent workability limitations of manufactured sand concrete.

The enhancement of mechanical properties in manufactured sand concrete primarily relies on two approaches: the incorporation of supplementary cementitious materials and the optimization of powder content. Stone powder, a byproduct generated during manufactured sand processing, improves aggregate gradation and enhances concrete densification. However, excessive amounts can adversely affect performance. Studies demonstrate that the compressive strength of manufactured sand concrete exhibits a characteristic trend—initially increasing and then decreasing—with rising powder content [12,13]. Under steam-curing conditions, this trend aligns with standard-cured specimens but shows accelerated strength degradation at higher powder concentrations [14,15]. When optimal workability and an appropriate powder dosage are maintained, the dual incorporation of fly ash and slag significantly enhances mechanical performance [8]. Building on this, Wang et al. employed mercury intrusion porosimetry and scanning electron microscopy to systematically investigate how powder content (by mass fraction) influences the microstructural characteristics of manufactured sand concrete and its subsequent impact on compressive strength [16,17]. These findings highlight the critical balance required in material composition to achieve optimal structural performance while mitigating the inherent limitations of manufactured sand concrete systems.

Current research on the durability of manufactured sand concrete predominantly focuses on the effects of powder and its methylene blue (MB) value, yet deeper insights into degradation mechanisms remain limited. The corrosion resistance coefficient of manufactured sand concrete decreases progressively with increasing powder content under identical wet–dry cycling conditions [18]. In steam-cured concrete systems, the powder content significantly influences capillary water absorption. Specimens with 10% powder exhibit the lowest capillary absorption coefficient and optimal impermeability [9,19]. Elevated MB values in powder correlate with reduced resistance to chloride ion penetration and carbonation [20]. Moreover, studies on mortar specimens containing gypsum minerals and a high powder content reveal severe sulfate erosion damage in low-temperature environments. This underscores the necessity for rigorous compositional testing and control when using manufactured sand as fine aggregate in tunnel concrete under such conditions [21,22]. These findings highlight the critical interplay between powder characteristics, environmental factors, and durability performance, emphasizing the need for tailored material design in manufactured sand concrete applications.

The distinct surface texture and morphological characteristics of manufactured sand fundamentally alter the microstructural formation behavior and interfacial properties of concrete compared with traditional natural river sand-based concrete, inevitably influencing its macroscopic performance. This study systematically investigates the fundamental characteristics of manufactured sand concrete under varying curing conditions, with a focused analysis on how standard curing and steam curing affect its static/dynamic mechanical properties, durability, deformation behavior, interfacial bonding quality, and pore structure characteristics across different parent rock-derived systems. This research specifically addresses critical challenges such as performance inconsistency, suboptimal interfacial adhesion, and insufficient durability in these concretes. By elucidating the underlying mechanisms of performance degradation and proposing improvement strategies, the findings establish a theoretical foundation for advancing eco-friendly construction materials. Furthermore, this study provides technical insights to support the expanded application of manufactured sand concrete in critical infrastructural components, bridging the gap between laboratory research and practical engineering implementation while promoting sustainable development in the construction industry.

## 2. Experimental Methodology

### 2.1. Materials and Mixing Proportions

#### 2.1.1. Raw Materials

(i) The Portland cement (P·O 42.5, PC) used in this study, produced by Hunan Pingtang Southern Cement Co., Ltd., Changsha, China, exhibits a density of 3.15 g/cm^3^ and a specific surface area of 367 m^2^/kg. Its experimentally determined mechanical strength parameters are summarized in Table 1, while its comprehensive physical properties and chemical composition are presented in Table 2, ensuring compliance with standard specifications for ordinary Portland cement applications.

(ii) The supplementary cementitious materials (SCMs) employed in this study comprised fly ash (FA) and ground granulated blast-furnace slag (GGBS), with their physicochemical properties detailed in Table 2. The FA, classified as Grade I according to Chinese National Standard GB/T 1596-2017 (fly ash used for cement and concrete) [23], exhibited a specific surface area of 450 m^2^/kg and a density of 2.4 g/cm^3^. The GGBS met the S95-grade requirements outlined in Chinese National Standard GB/T 18046-2017 (ground granulated blast-furnace slag used for cement, mortar and concrete) [24], demonstrating a specific surface area of 440 m^2^/kg and a density of 2.85 g/cm^3^, ensuring compliance with industrial standards for enhanced concrete performance.

**Table 2:** Composition of cementitious materials (wt%) [25].

Group	SiO_2_	Al_2_O_3_	Fe_2_O_3_	CaO	MgO	SO_3_	eq-Na_2_O	LOI
PC	24.6	7.3	4.0	59.7	3.8	2.5	0.6	2.5
FA	52.3	26.3	9.7	3.7	1.2	0.2	1.8	5.7
GGBS	26.1	13.8	14.1	33.6	8.1	\	0.45	2.1

(iii) The manufactured sands utilized in this investigation consisted of granite-based (S1) and limestone-based (S2) varieties, supplied by the Hunan Branch of China West Construction Group Co., Ltd., Changsha, China. These sands were produced using industrial vertical shaft impact crushers, simulating real-world production conditions rather than laboratory-scale crushing. These sands were employed in their as-produced state without water washing to replicate practical concrete batching plant conditions, exhibiting fineness moduli of 2.4 and 2.5, respectively. According to the crushing condition of the manufactured sand and the manufacturer’s warranty, it is known that S1 sand contains a certain amount of mica minerals; moreover, the MB value is within the acceptable range for concrete production. The manufactured sands are shown in Figure 1. The coarse aggregates (G) comprised continuously graded limestone particles (5–20 mm), with a controlled particle size distribution—40% of the 5–10 mm fraction and 60% of the 10–20 mm fraction—ensuring optimal packing density and mechanical interlock characteristics for enhanced concrete performance.

(iv) The superplasticizer (SP) employed in this study was PCA-I polycarboxylate-based high-performance water reducer produced by Subote New Materials Co., Ltd., Nanjing, China, characterized by its optimal dispersion efficiency and dosage-dependent workability enhancement. The mixing water (W) consisted of standard tap water meeting ASTM C1602 requirements for concrete production [26].

#### 2.1.2. Mixing Proportions

The mixing proportions for all manufactured sand concretes are shown in Table 3.

Specifically, G1 denotes the group where limestone-manufactured sand is completely replaced by granite-manufactured sand; G2 serves as the control group (100% limestone-manufactured sand); and G3 represents a partial replacement mixture (50% granite + 50% limestone).

### 2.2. Specimen Preparation and Curing Schemes

The concrete preparation followed standardized procedures using a forced-action mixer, commencing with pre-treatment where mixing blades and bowl surfaces were conditioned with trial-mix mortar equivalent to 5% of the total batch weight to minimize material adhesion. After mortar discharge, aggregates were dry-mixed for 30 s prior to cementitious materials’ incorporation and subsequent 30 s of homogenization. Liquid components (water and admixture) were then introduced within a strict period of 2 min, followed by a 2 min final mixing phase ensuring a homogeneous distribution. The fresh concrete exhibited controlled slump values of 50–80 mm prior to mold casting, with subsequent vibration compaction achieving optimal void reduction and structural integrity, maintaining compliance with ASTM C192/C192M mixing and curing specifications [27].

**Figure 2:**
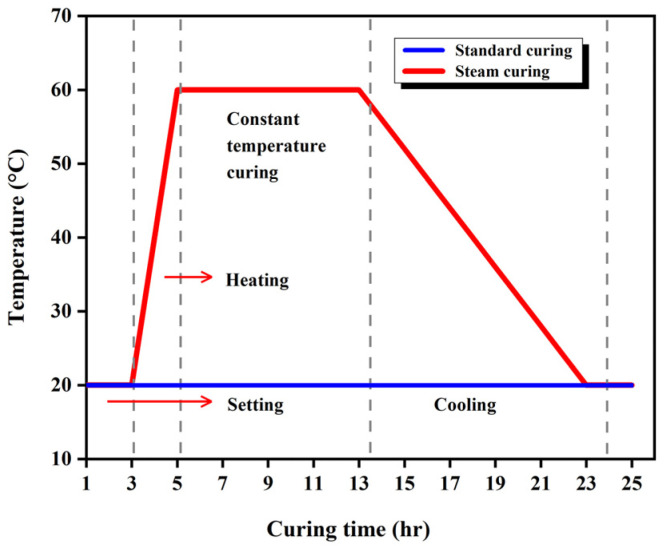
Designed curing regimes for manufactured sand concrete.

The specimens were subjected to dual curing protocols: standard curing and steam curing. Immediately after casting, all specimens were maintained in a controlled environment (23 ± 2 °C; 40–50% *RH*), with surface-sealing polyethylene membranes to prevent moisture loss. For standard-cured specimens, demolding occurred at 24 h of maturity, followed by transfer to a standard curing room (20 ± 2 °C; ≥95% *RH*) until testing ages. Concurrently, a concrete quick-curing box was used to implement steam curing. During the heating and constant-temperature stages, the relative humidity inside the box was ≥95% *RH*. Steam-cured specimens underwent a three-phase thermal treatment: 3 h of pre-curing under ambient conditions, followed by 2 h of heating to 60 ± 1 °C, 8 h of isothermal maintenance, and 2 h of controlled cooling to room temperature. The post-thermal treatment specimens were demolded and subsequently stored under standard-curing conditions, ensuring comparative analysis validity between curing methodologies. This dual-regime approach enabled systematic evaluation of hydration kinetics and microstructure development under conventional versus accelerated curing conditions. The curing regimes for concrete are shown in Figure 2.

### 2.3. Test Methods

#### 2.3.1. Mechanical Property

(i) Compressive and splitting tensile strength evaluations were conducted in accordance with Chinese National Standard GB/T 50081-2019 (standard for test methods of concrete’s physical and mechanical properties) [28], utilizing 100 mm cubic specimens in triplicate sets. Load application rates were strategically differentiated across curing periods: 0.6 MPa/s for 7 d specimens versus 0.8 MPa/s for 28 d and 56 d specimens, accommodating strength development characteristics. Raw compressive strength values were multiplied by a 0.95 conversion factor prior to averaging, while splitting tensile results underwent 0.85 scaling, aligning with standardized protocols for dimensional correction and interfacial stress distribution normalization.

(ii) Dynamic elastic modulus measurements were performed using the Emodule-Meter Mk II resonant frequency meter on triplicate 100 mm × 100 mm × 300 mm concrete prism specimens. Longitudinal mode testing was conducted with optimized parameters: the signal gain set to 25, a sampling rate of 40 Hz, and 1024-point data acquisition. Specimens were mechanically excited through controlled impacts using a calibrated impact hammer on axial surfaces, while an integrated accelerometer recorded the resulting acceleration decay waveforms. Testing was performed at 7 d, 28 d, and 56 d curing intervals. Post-measurement, the system automatically computed the fundamental longitudinal frequency via fast Fourier-transform (FFT) analysis of the attenuation curves and derived dynamic elastic modulus values using Equation (1).

(1) $$E_{d}=D\cdot M\cdot {\left(n_{1}\right)}^{2}$$

where *E_d_* represents the dynamic elastic modulus (GPa); *D* is the geometric parameter calculated as *D* = 4·(*L*/*b*·*t*); m is the weight of the specimen (kg); and *n*_1_ represents the fundamental longitudinal frequency (Hz).

(iii) The damping ratio quantifies vibration energy dissipation within concrete’s microstructural framework, serving as a critical indicator of internal friction and crack propagation resistance [29,30]. In this study, the parameter was determined through spectral analysis of acceleration decay profiles captured by the resonant frequency analyzer. Employing the free vibration decay method [31,32,33], damping ratios were calculated via Equation (2).

(2) $$\xi =\frac{1}{2n\pi }\cdot \ln\frac{A_{1}}{A_{2}}$$

where *ξ* is the damping ratio; *A*_1_ and *A*_2_ represent consecutive amplitude peaks in the acceleration decay curve, with *n* denoting the number of oscillation cycles between them. Selecting *n* values of 50, 60, or 70 between consecutive peaks yielded stable damping ratio calculations.

#### 2.3.2. Deformation Property

**Figure 3:**
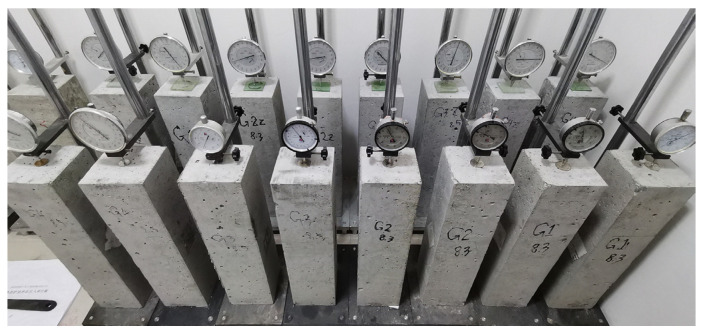
Dry shrinkage deformation test of manufactured sand concrete.

Drying shrinkage deformation *ε_dry_* was quantified using a vertical shrinkage frame with the linear measurement method (accuracy: 0.01 mm) on triplicate 100 mm × 100 mm × 400 mm concrete prism specimens, with initial readings (*L*_0_) recorded at 1 d of standard curing and post-steam-curing demolding, followed by periodic measurements (*L_t_*) under controlled laboratory conditions (23 ± 2 °C; 60 ± 5% *RH*) according to Equation (3), as illustrated in Figure 3.

(3) $$\varepsilon_{dry}=\frac{L_{0}-L_{t}}{L_{0}}$$

#### 2.3.3. Durability

(i) The capillary absorption evaluation was conducted in accordance with ASTM C642-13 (standard test method for density, absorption, and voids in hardened concrete) using the initial surface absorption test (ISAT) method [34,35,36,37,38,39,40,41]. Three 100 mm × 50 mm concrete disc specimens were tested to measure water mass variation over time across a defined surface area, with capillary absorption performance quantitatively characterized through Equations (4) and (5).

(4) $$i=A+S\cdot t^{0.5}$$

(5) $$i=\frac{\Delta W}{A_{r}\cdot \rho_{0}}$$

where the absorption rate (*i*) represents cumulative water absorption per unit area (mm); time (*t*) is measured in minutes; Δ*W* is the water absorption mass (g); *A_r_* is the cross-sectional area of the specimen (mm^2^); *ρ*_0_ is the density of water (g/cm^3^); *A* serves as a constant; and *S* is the capillary adsorption coefficient (mm/min^1/2^), representing the permeation rate of capillary rise.

Following a 56 d curing period, specimens underwent 48 h of oven drying at 60 °C to achieve complete initial desiccation. Each specimen was perimeter-sealed with a silicone sealant to enforce single-surface water absorption and then partially immersed (≤5 mm depth) with continuous water level monitoring. Mass measurements were conducted at 0 min, 1 min, 5 min, 10 min, 20 min, 30 min, 1 h, 2 h, 3 h, 4 h, 5 h, 6 h, 12 h, 1 d, 2 d, 3 d, 4 d, 5 d, 6 d, and 7 d using a 0.1 g precision electronic balance. Surface moisture was blotted prior to weighing, with each measurement completed within 15 s. Triplicate specimens were tested with results averaged for data reliability.

(ii) Chloride ion permeability testing was performed following the electric flux method specified in Chinese National Standard GB/T 50082-2024 (standard for test methods of long-term performance and durability of ordinary concrete) [42], with measurements conducted at 56 d of curing maturity.

#### 2.3.4. Interfacial Property

**Figure 4:**
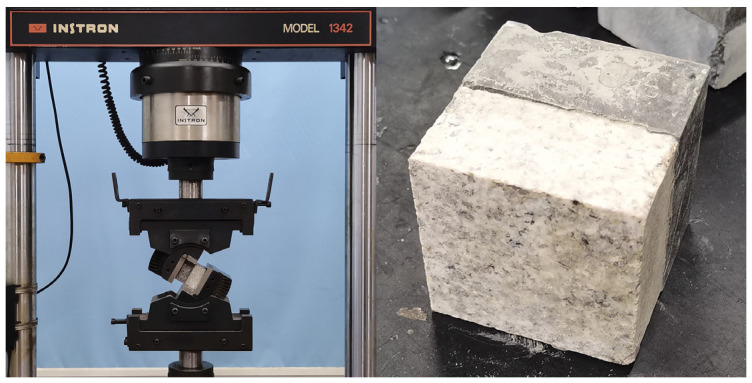
Variable-angle shear test of manufactured sand parent rock and hardened cement composite specimen.

**Figure 5:**
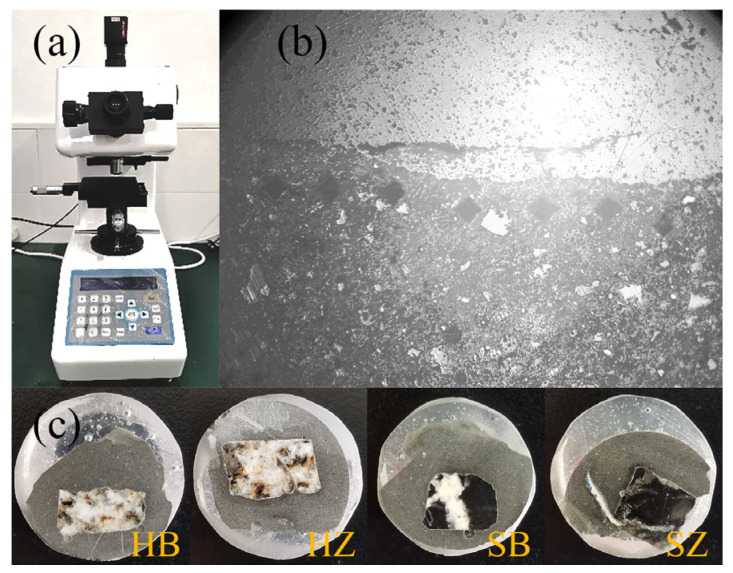
Microhardness test: (**a**) tester; (**b**) test results; and (**c**) test specimens.

(i) The interfacial shear strength evaluation was conducted using an Instron 1342 apparatus for variable-angle shear testing at a loading rate of 0.12 mm/min, with the experimental setup and specimen configuration detailed in Figure 4. Triplicate composite specimens (56 d curing) comprising 50 mm × 50 mm × 25 mm manufactured sand parent rock blocks bonded to hardened cement paste blocks were tested at inclination angles of 50°, 60°, and 70°. The results were calculated using Equation (6).

(6) τ=c+σ⋅tanϕ

where *τ* is the peak shear stress; *σ* is the normal stress; *c* is the cohesion; and *φ* represents the internal friction angle.

(ii) The interface transition zone properties were evaluated through microhardness testing on 56 d cured specimens. After hydration termination via ethanol immersion (stored in anhydrous ethanol for 7 days with solution replacement after 24 h), the specimens were vacuum-dried for ≥2 days with a desiccant. Surface preparation involved 1200-grit sandpaper grinding (15 min minimum), ultrasonic cleaning in isopropanol, and epoxy resin impregnation with bubble removal. After 24 h of curing at room temperature, specimens underwent additional 1200-grit grinding to expose surfaces. Multi-stage polishing (5 μm → 2.5 μm → 1 μm metallographic compounds; 30 min each) preceded ultrasonic cleaning between stages [43,44,45]. The ITZ between cement paste and manufactured sand parent rock was analyzed using an MC010 microhardness analysis system. Key testing parameters included an applied load of 0.0981 N and a dwell time of 15 s. Indentation points were systematically selected along aggregate boundaries during ITZ microhardness testing, with calculated values derived using Equation (7).

(7) $$HV=0.102\times \frac{F}{S}=0.102\times 2\times F\cdot \frac{\sin(\theta /2)}{d^{2}}=0.1891\times \frac{F}{d^{2}}$$

where *HV* denotes the Vickers hardness; *F* represents the applied load; *S* corresponds to the indentation area; *d* signifies the arithmetic mean of indentation diagonals; and *θ* indicates the indenter face angle. The microhardness tester and test results are shown in Figure 5a and Figure 5b, respectively, with the test results magnified 200 times. Figure 5c displays the ITZ microhardness specimen between manufactured sand parent rock and hardened cement paste. HB and HZ denote standard-cured and steam-cured granite–cement paste composite specimens, while SB and SZ represent standard-cured and steam-cured limestone–cement paste composite specimens.

#### 2.3.5. Pore Structure

Low-field nuclear magnetic resonance (NMR) testing can directly reflect changes in pore size distribution within hardened cement-based materials. The test was conducted using a China SUZHOU NIUMAG 2 MHz NMR system (MicroMR02-050V, LIMECHO, Suzhou, China) to characterize the pore structure of the concrete core samples. The built-in program of the equipment was used for testing, maintaining the magnet temperature at 35 °C. After a series of debugging and calibration steps, the *T*_2_ spectrum sequence was applied, and key parameters were set in the CPMG test interface: the echo time was set to 100μs, the reply time was set to 1s, the echo count was set to 5000, and the scan was set to 128. The testing principle is as follows [46,47]: In a water-saturated state, the transverse relaxation rate 1/*T*_2_ of fluid in pores is the sum of free relaxation 1/*T*_2*B*_, surface relaxation 1/*T*_2*S*_, and diffusion relaxation 1/*T*_2*D*_. *T*_2*B*_ represents the transverse relaxation time of fluid in bulk containers (negligible), *T*_2*S*_ is the transverse relaxation time caused by surface relaxation, and *T*_2*D*_ is the transverse relaxation time induced by diffusion under magnetic field gradients (also negligible). Therefore, the transverse relaxation rate 1/*T*_2_ can be expressed by Equation (8).

(8) $$\frac{1}{T_{2}}=\frac{1}{T_{2B}}+\frac{1}{T_{2S}}+\frac{1}{T_{2D}}\approx \frac{1}{T_{2S}}=\rho_{2}\cdot \frac{S}{V}$$

where *ρ*_2_ represents the surface relaxation rate, and the *S*/*V* is the specific surface area of pores. Therefore,

(9) $$r=\rho_{2}\cdot T_{2}\cdot a$$

It follows that the pore radius r of porous materials is proportional to T2; thus, the T2 spectrum reflects the pore size distribution of the sample. In this study, ρ2 was set to 0.05 μm/ms and a to 2 (cylindrical model). The water-saturated specimen volume was calculated using the liquid displacement method (deionized water). The water saturation process involved placing specimens in a vacuum saturation apparatus, evacuating for 3 h, injecting deionized water under vacuum to submerge the specimens, maintaining submersion for 3 h after vacuum release, followed by 3 h of ambient-pressure immersion. The testing age was 56 d.

## 3. Results and Discussion

### 3.1. Static and Dynamic Mechanical Properties

#### 3.1.1. Compressive Strength

**Figure 6:**
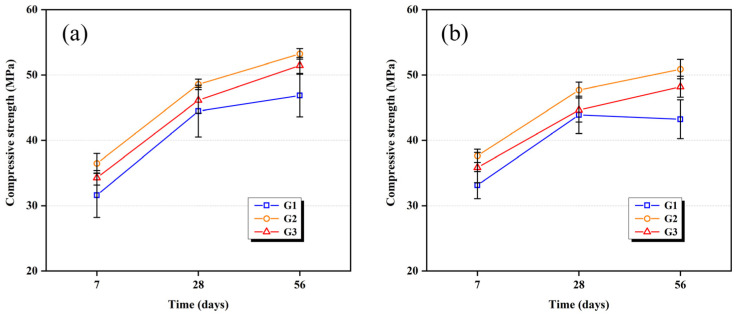
Test results of compressive strength test of manufactured sand concrete: (**a**) standard-curing condition; (**b**) steam-curing condition.

The compressive strength test results of manufactured sand concrete with different rock types under different curing conditions are shown in Figure 6.

As shown in Figure 6, under both standard-curing and steam-curing conditions, the compressive strength of granite-manufactured sand concrete (group G1) was the lowest at all ages, while limestone-manufactured sand concrete (group G2) exhibited the highest compressive strength. The compressive strength of concrete with 50% granite-manufactured sand (group G3) fell between the two. At 7 d, the steam-cured groups generally showed higher compressive strength than the standard-cured groups. However, by 28 d, none of the groups achieved 50 MPa under either curing condition, with steam-cured concrete slightly underperforming standard-cured concrete. This is attributed to reduced cement content due to mineral admixtures and thermal damage in steam-cured concrete [48]. At 56 d, groups G2 and G3 under standard curing exceeded 50 MPa, reaching approximately 53 MPa and 51.5 MPa, respectively. Under steam curing, only group G2 reached about 51 MPa, indicating unavoidable internal damage from steam curing in the manufactured sand concrete. Additionally, group G1 under steam curing showed strength regression compared with standard curing, highlighting the significant influence of granite-manufactured sand on compressive strength. However, partial replacement of limestone-manufactured sand with granite-manufactured sand still improved compressive strength to some extent. The compressive strength difference between group G3 and group G2 under both curing conditions was only 3–6%, with stable strength growth over time. The observed strength variations primarily stem from the mica content in the granite-manufactured sand used in this batch. Flaky mica weakens the ITZ between cement paste and aggregates, accelerating performance degradation [49,50,51]. Despite this, based on the compressive strength results, further reducing the granite-manufactured sand content and optimizing steam-curing parameters could enable the production of C50-grade concrete containing granite-manufactured sand.

#### 3.1.2. Splitting Tensile Strength

**Figure 7:**
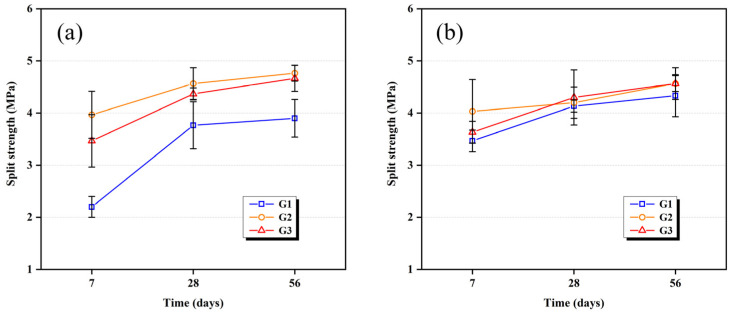
The results of splitting tensile strength test of manufactured sand concrete: (**a**) standard-curing condition; (**b**) steam-curing condition.

The splitting tensile test results of manufactured sand concrete with different rock types under different curing conditions are shown in Figure 7.

As shown in Figure 7, under both standard-curing and steam-curing conditions, the splitting tensile strength of group G1 was the lowest at all ages, while group G2 maintained nearly the highest splitting tensile strength. Although group G3 fell between the two, its splitting tensile strength was generally closer to that of group G2. Standard curing was most effective in improving the 28-day splitting tensile strength of concrete, especially that of group G1, which increased by approximately 71% from 2.2 MPa at 7 d to around 3.8 MPa, showing the fastest growth rate among all groups. However, its effectiveness in enhancing 56 d splitting tensile strength was limited. At 7 d, except for the standard-cured group G1, the splitting tensile strength of other groups ranged between 3.5 and 4.0 MPa, and steam curing improved the splitting tensile strength of all groups. At 28 d, under standard curing, group G1 slightly exceeded group G3 but remained below 4.0 MPa, while under steam curing, the splitting tensile strengths of all groups were similar, at around 4.2 MPa. By 56 d, both curing methods showed limited improvement in splitting tensile strength for all groups, but groups G2 and G3 still maintained values around 4.7 MPa. The observed patterns in splitting tensile strength align with previous compressive strength trends, but the splitting tensile results more clearly demonstrate the adverse effects of mica in granite-manufactured sand on concrete’s mechanical performance. Mica’s brittle nature may induce fragile failure in concrete, reducing tensile performance, particularly in high-strength concrete. Mica particles are prone to fracture, promoting crack propagation and lowering tensile strength [52]. A higher mica content likely exacerbates these negative impacts on splitting tensile strength.

#### 3.1.3. Dynamic Modulus of Elasticity

**Figure 8:**
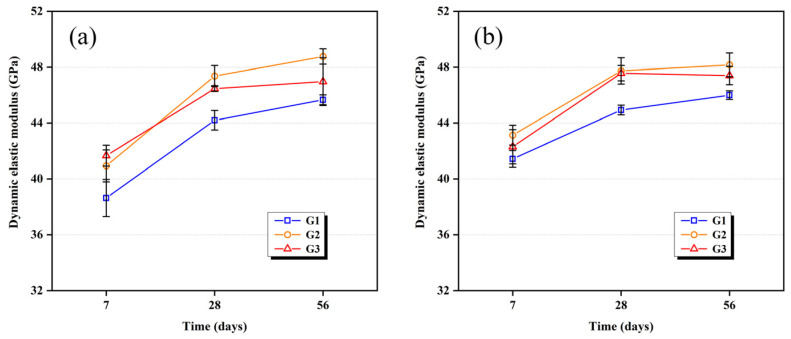
The results of dynamic modulus of elasticity test of manufactured sand concrete: (**a**) standard-curing condition; (**b**) steam-curing condition.

The dynamic modulus of elasticity test results of manufactured sand concrete with different rock types under different curing conditions are shown in Figure 8.

As shown in Figure 8, the variation patterns of the dynamic elastic modulus for all concrete groups under standard-curing and steam-curing conditions were generally consistent with the compressive strength and splitting tensile strength test results. At 7 d, under standard curing, the dynamic elastic modulus of groups G2 and G3 was around 41 GPa, while only that of group G1 remained below 40 GPa. Under steam curing, however, the dynamic elastic modulus of all groups increased to approximately 42 GPa, indicating that the steam-curing regime, despite causing inevitable thermal damage to concrete, could promote the rapid development and formation of internal microstructures at early stages. By 28 d, the dynamic elastic moduli of all groups generally ranged between 44 and 48 GPa, but that of group G1 still did not exceed 45 GPa, with even lower enhancement from steam curing. This clearly demonstrates, as previously discussed, that internal defects caused by mica in granite-manufactured sand could no longer be compensated by long-term hydration product filling. At 56 d, the dynamic elastic modulus development of groups G1 and G3 became extremely slow, yet that of group G3 reached around 47.5 GPa under both curing conditions, significantly higher than group G1. Meanwhile, steam curing further exacerbated the continuous deterioration of internal structures and performance in granite-manufactured sand concrete, which might explain the compressive strength regression in group G1. Reducing the granite-manufactured sand content still significantly improved concrete’s dynamic elastic modulus. Group G2 exhibited the highest dynamic elastic modulus at 56 d, exceeding 48 GPa under both curing conditions, demonstrating superior internal structural integrity in limestone-manufactured sand concrete compared with granite-manufactured sand concrete.

#### 3.1.4. Damping Ratio

**Figure 9:**
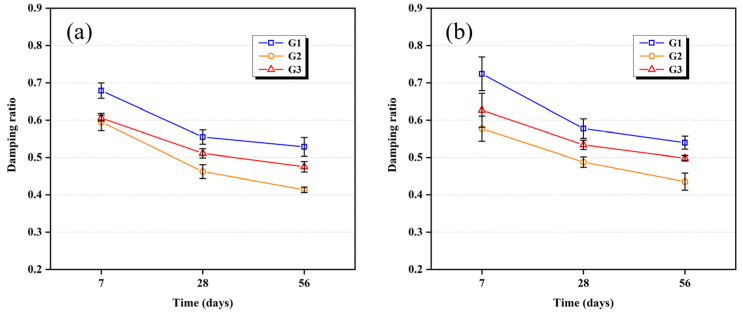
The results of damping ratio calculation of manufactured sand concrete: (**a**) standard-curing condition; (**b**) steam-curing condition.

The damping ratio calculation results of manufactured sand concrete with different rock types under different curing conditions are shown in Figure 9.

From Figure 9, the damping ratio calculation results more realistically reflect the internal structural conditions of each concrete group, particularly in terms of energy dissipation. At all curing ages, the damping ratios of the concrete groups under steam-curing conditions are generally significantly higher than those under standard-curing conditions. This demonstrates the deterioration effect of steam curing on concrete materials, especially regarding the coarsening of pore structures and the formation of internal microcracks, while also revealing the adverse effects of granite-manufactured sand incorporation on the formation of microstructural features. At 7 d, group G1 under steam curing exhibited the highest damping ratio (exceeding 0.7) among all groups. However, with a reduced granite-manufactured sand content, group G3 showed a rapid decline in its damping ratio to approximately 0.6, even approaching that of group G2 under standard curing, indicating that decreasing the granite-manufactured sand content improves the microstructural development of manufactured sand concrete. By 28 d, only group G2 exhibited damping ratios below 0.5 under both curing conditions, while that of group G1 remained elevated. At 56 d, all groups show continued but decelerated damping ratio reduction. Notably, group G1 demonstrated comparable damping ratios (approximately 0.53) between curing conditions despite having the lowest dynamic elastic modulus, suggesting probable compressive strength regression. Meanwhile, group G3 achieved damping ratios below 0.5, confirming enhanced microstructural refinement. Group G2 under standard curing showed the lowest damping ratio (approaching 0.4) among all groups.

The above results indicate that concrete prepared with single-limestone-manufactured sand exhibits a denser internal microstructure, where the thermal damage caused by the steam-curing regime is primarily attributed to the performance degradation of manufactured sand concrete.

### 3.2. Drying Shrinkage Behavior

**Figure 10:**
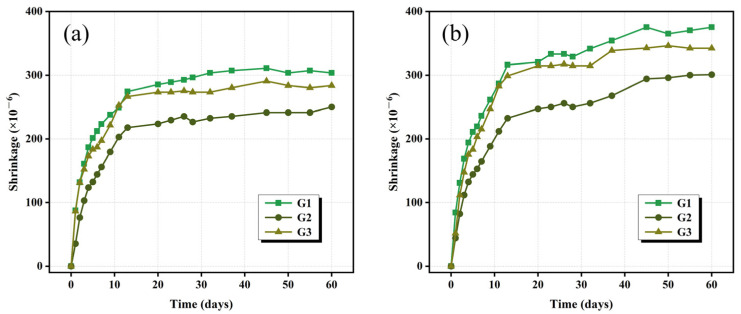
The results of drying shrinkage test of manufactured sand concrete: (**a**) standard-curing condition; (**b**) steam-curing condition.

The drying shrinkage results of manufactured sand concrete with different rock types under different curing conditions are shown in Figure 10.

As shown in Figure 10, the drying shrinkage deformation of all concrete groups exhibited rapid growth within 14 days under both standard-curing and steam-curing conditions, gradually slowing by 28 days. However, the increase in drying shrinkage deformation under steam curing was significantly higher than that under standard curing. Group G2 demonstrated the lowest drying shrinkage deformation throughout the testing period, indicating that concrete prepared with single-limestone-manufactured sand possessed refined pore structures, fewer internal defects from raw materials, and reduced open pores/pathways for moisture migration. At 60 d, group G2 showed drying shrinkage deformations of approximately 250 με and 300 με under standard curing and steam curing, respectively, representing a 20% increase. This discrepancy primarily resulted from steam curing coarsening the pore structure of manufactured sand concrete and promoting microcrack propagation, which substantially increased open pores and connected pathways. Group G1 exhibited the highest drying shrinkage deformation, reaching approximately 300 με and 390 με under standard curing and steam curing at 60 days (30% increase). This was mainly attributed to the inferior microstructure of granite-manufactured sand concrete, particularly aggravated by steam curing, which reduced resistance to volumetric deformation and accelerated microcrack development and pore connectivity [53], thereby intensifying moisture loss in drying shrinkage tests. Group G3 consistently showed 10–30 με lower deformation than group G1 after 14 days under both curing conditions, suggesting a reduced granite-manufactured sand content could inhibit drying shrinkage.

While previous studies indicated reduced granite content improved static/dynamic mechanical properties compared with full-granite-manufactured sand concrete, group G3 still showed 280–350 με drying shrinkage at 60 days, potentially compromising volumetric stability. This implies the need for a further reduction in the granite content or alternative methods to enhance the microstructures of the G1 and G3 groups.

### 3.3. Durability

#### 3.3.1. Water Absorption

**Figure 11:**
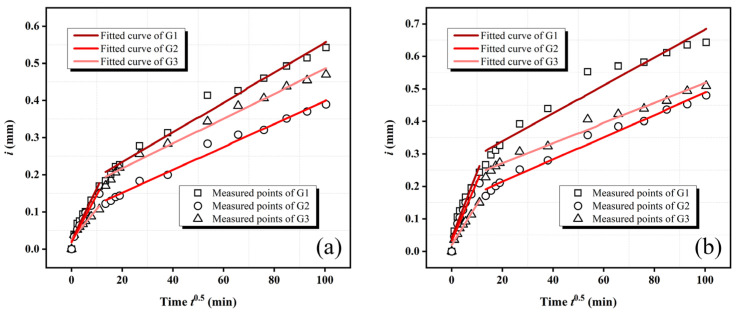
The results of capillary water absorption test of manufactured sand concrete: (**a**) standard-curing condition; (**b**) steam-curing condition.

Capillary water absorption is the primary ion transport mechanism in concrete, directly affecting its durability, such as frost resistance, chloride ion penetration resistance, and sulfate attack resistance. The water absorption results of manufactured sand concrete with different rock types under different curing conditions are shown in Figure 11. The fitting results of the capillary adsorption coefficient are shown in Table 4.

According to Figure 11, the capillary adsorption rate of all concrete groups increased rapidly within 2 h. From 2 h to 7 d, the capillary adsorption rate continued to rise but showed a significantly slower growth trend. Notably, the capillary adsorption rates of all groups under steam-curing conditions were generally higher than those under standard-curing conditions. Regardless of curing conditions, group G2 consistently exhibited the lowest capillary adsorption rate, while group G1 maintained the highest rate. These observations further validate previous analyses of granite-manufactured sand’s impact on concrete’s internal microstructure and the effects of steam-curing regimes on manufactured sand concrete performance, as discussed from the perspectives of static/dynamic mechanical properties and deformation characteristics.

Specifically, according to Table 4, the capillary absorption rates of concrete with different lithology manufactured sands showed excellent fitting under various curing conditions. Within 2 h, under standard-curing conditions, the capillary absorption rate of group G2 was 0.0084 mm/min^0.5^, while those of both groups G1 and G3 exceeded 0.01 mm/min^0.5^, with that of group G1 reaching 0.0141 mm/min^0.5^, nearly 1.8 times that of group G2. Steam curing further increased the capillary absorption rates of all groups, with that of group G2 reaching 0.0125 mm/min^0.5^ (approximately 50% higher than that under standard curing). Meanwhile, group G1 exhibited a rapid rise exceeding 0.02 mm/min^0.5^. Over longer testing periods, all groups showed decreased capillary absorption rates, though group G1 maintained the highest levels under both standard (0.0040 mm/min^0.5^) and steam curing (0.0043 mm/min^0.5^). Notably, under standard curing, group G2 displayed lower absorption rates than group G3, while under steam curing, this relationship reversed. These variations may be influenced by multiple factors, including improper specimen formation and inadequate bonding between silicone rubber and concrete.

#### 3.3.2. Resistance to Chloride Ion Permeability

**Figure 12:**
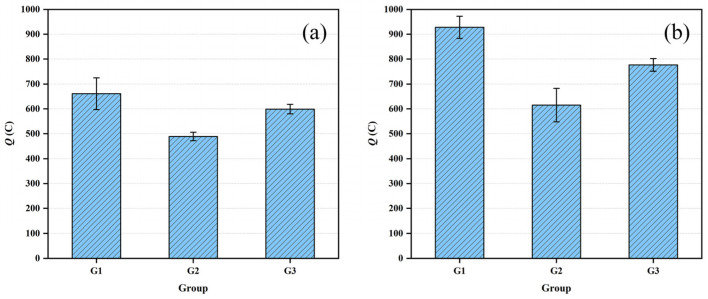
The results of electrical flux test of manufactured sand concrete: (**a**) standard-curing condition; (**b**) steam-curing condition.

According to the ASTM C1202-97 DC electrical flux method [54], the chloride ion permeability of concrete can be classified into four grades based on the coulomb charge passed through the sample: “high” (>4000 C), “moderate” (2000~4000 C), “low” (1000~2000 C), “very low” (100~1000 C), and “negligible” (<100 C). The electrical flux test results of manufactured sand concrete with different rock types under different curing conditions are shown in Figure 12.

As shown in Figure 12, the electric flux of all concrete groups remained below 1000 C, indicating the good chloride ion penetration resistance of manufactured sand concrete at this strength grade. The electric flux test results align closely with the capillary water absorption test results. Under standard-curing conditions, the electric flux of group G2 was below 500 C, while those of groups G1 and G3 both exceeded 600 C. Under steam-curing conditions, the electric flux of G2 slightly increased to approximately 610 C compared with standard curing, whereas groups G1 and G3 showed more significant increases. The maximum electric flux reached approximately 920 C for G1 (a 40% increase from standard curing) and nearly 800 C for G3 (a 30% increase). This demonstrates that the introduction of granite-manufactured sand combined with a steam-curing regime reduces the durability performance of concrete.

### 3.4. Interfacial Property

#### 3.4.1. Interfacial Shear Strength

**Figure 13:**
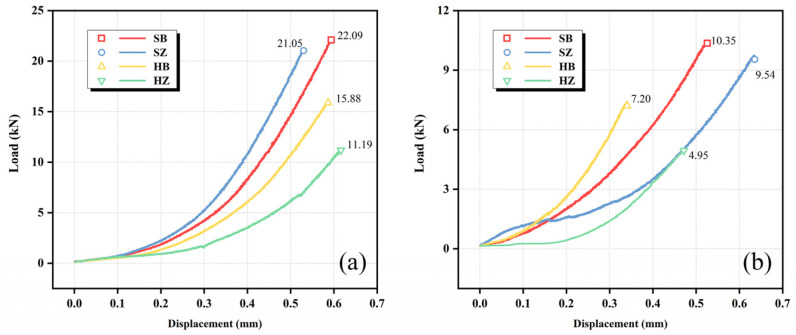
The results of shear strength test at different angles of manufactured sand parent rock and hardened cement paste: (**a**) 50°; (**b**) 60°.

During the experimental process, the angle of variable-angle shear testing for manufactured sand concrete plays a crucial role in determining the test results and their reliability. When testing at a large angle of 70°, most specimens experienced interface shear failure while still in the pre-compaction phase or during the early stages of formal testing. The load data collected under these conditions exhibited significant discrepancies and errors. Therefore, this study excludes test data obtained from variable-angle shear tests conducted at 70° and focuses solely on analyzing experimental data acquired at 50° and 60° angles. The shear strength test results of manufactured sand concrete with different rock types under different curing conditions are shown in Figure 13. SB and SZ represent standard-cured and steam-cured limestone–cement paste composite specimens, respectively, while HB and HZ denote standard-cured and steam-cured granite–cement paste composite specimens, respectively.

**Figure 14:**
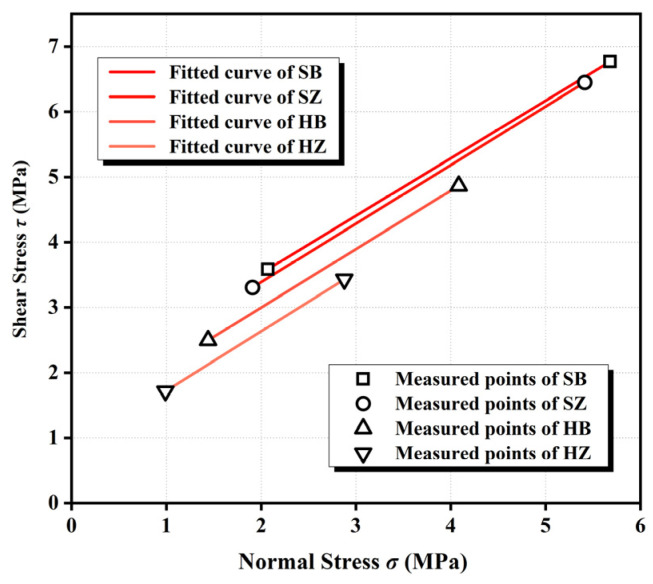
Shear stress fitting results of manufactured sand parent rock and hardened cement paste.

As shown in Figure 13, under standard-curing and steam-curing conditions, the peak loads of limestone–cement paste composite specimens at 50° and 60° angles were relatively close, measuring approximately 21.5 kN and 10 kN. respectively. Steam curing reduced the peak load by 4.7–7.8%. A 10° increase in the testing angle caused a drastic decline in the peak load, with reductions of approximately 53.1% and 54.7% for standard-cured and steam-cured limestone–cement paste specimens, respectively, indicating a significant change in stress patterns. Granite–cement paste specimens exhibited substantially lower peak loads across all angles compared with their limestone counterparts, with maximum and minimum values of 15.88 kN (standard curing) and 4.95 kN (steam curing). Their peak load reduction rate with increasing angles was approximately 30%, lower than that of the limestone specimens. At 50°, the displacements of all composite specimens ranged from 0.5 to 0.6 mm. At 60°, the limestone specimens maintained relatively high displacements, while the granite specimens failed at displacements of 0.35–0.45 mm. These results reflect the weaker interfacial bonding between granite and cement paste compared with limestone. Figure 14 shows the shear stress fitting results for different lithology aggregates and hardened cement paste under various curing conditions, with the corresponding shear strength parameters listed in Table 5.

Cohesion is typically related to the chemical properties and microstructure of materials, while the internal friction angle is associated with the physical shape and surface characteristics of particles. In this study, cohesion reflects the bonding condition between manufactured sand parent rock and hardened cement paste interface, which correlates with intermolecular forces and the interfacial microstructure [55,56].

As shown in Figure 14 and Table 5, under standard-curing conditions, the limestone–cement paste composite specimens exhibited the highest cohesion among all groups, reaching 1.76 MPa, approximately 46.7% higher than that of granite–cement paste composite specimens. Under steam-curing conditions, the granite–cement paste composite specimens showed the lowest cohesion (below 1.0 MPa). Additionally, steam-cured composite specimens exhibited lower cohesion compared with standard-cured ones, with reductions of approximately 9.66% and 32.5% for limestone–cement paste and granite–cement paste composites, respectively. These results indicate that steam curing minimally affects the interfacial performance of limestone–cement paste composites but exacerbates interface degradation in granite–cement paste composites. This discrepancy arises because (i) the mismatch in thermal expansion coefficients between cement paste and parent rocks induces significant thermal deformation and temperature-induced stresses during heating and cooling stages, leading to weaker interfacial bonding [57]; (ii) variations in intermolecular forces and interfacial air void contents between different parent rocks and cement paste result in divergent cohesion values.

Notably, the internal friction angle of all composite specimens gradually increased, likely influenced by the surface roughness, particle shape, and microstructural features of the parent rocks. The calculated results demonstrate that granite exhibits higher surface roughness than limestone, attributed to its irregular cracks and textured surfaces formed by minerals such as feldspar, quartz, and mica.

#### 3.4.2. ITZ Property

**Figure 15:**
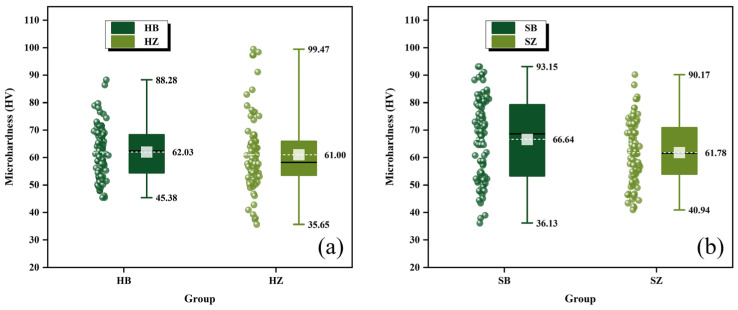
Microhardness test results of ITZ between manufactured sand parent rock and hardened cement paste: (**a**) granite–cement paste interface; (**b**) limestone–cement paste interface.

**Figure 16:**
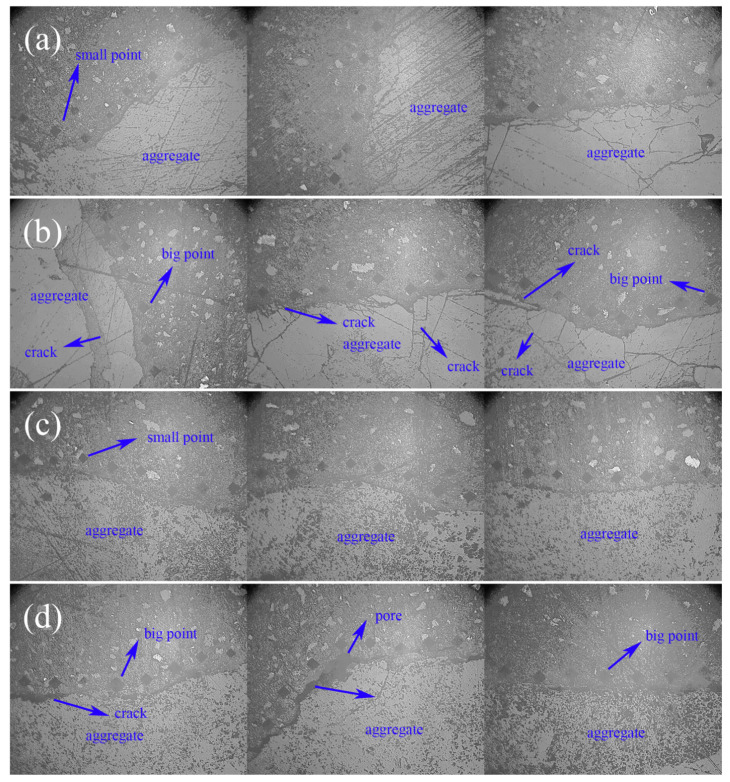
Microhardness indentation marks in ITZ between manufactured sand parent rock and hardened cement paste: (**a**) HB; (**b**) HZ; (**c**) SB; and (**d**) SZ.

The microhardness test results and indentation characteristics of the ITZ between manufactured sand parent rocks of different lithologies and hardened cement paste under different curing conditions are shown in Figure 15 and Figure 16, respectively.

As shown in Figure 15, under both standard-curing and steam-curing conditions, the average interfacial microhardness of limestone–cement paste composite specimens was higher than that of granite–cement paste composite specimens, and the steam-curing regime reduced the average interfacial microhardness between manufactured sand parent rock and hardened cement paste. Specifically, the limestone–cement paste composite specimens under standard curing exhibited the highest average interfacial microhardness at 66.64 *HV*, approximately 7.4% higher than that of granite–cement paste composite specimens. Under steam curing, the average values of both types were similar, with the granite–cement paste composite specimens showing the lowest average interfacial microhardness at 61 *HV*. The microhardness test results validate the interfacial shear strength measurements, particularly supporting the calculated cohesion values.

Figure 16 reflects the surface characteristics of manufactured sand parent rocks and the features of the ITZ. On the one hand, the hardened cement paste contains some unhydrated cement clinker, slag, and occasionally fractured fly ash particles. The cement paste appears dense without visible bubbles, pores, or cracks. The limestone surface remains smooth with no significant fractures or fragmentation, nor loose particles. In contrast, the granite surface shows noticeable polishing marks along with prominent fractures and fragmentation, even containing loose crushed stones embedded in the hardened cement paste, which may primarily contribute to the larger internal friction angle of granite–cement paste composite specimens. On the other hand, regarding indentation marks, under standard-curing conditions, the diameters of indentation points created by the microhardness tester at the ITZ between manufactured sand parent rocks and hardened cement paste are generally smaller than those under steam curing. This indicates that steam curing weakens the performance of the ITZ, resulting in looser bonding interfaces between cement paste and manufactured sand parent rocks. This phenomenon may correlate with increased microcrack formation and coarsened pore structures [58].

### 3.5. Pore Structure Characteristics

**Figure 17:**
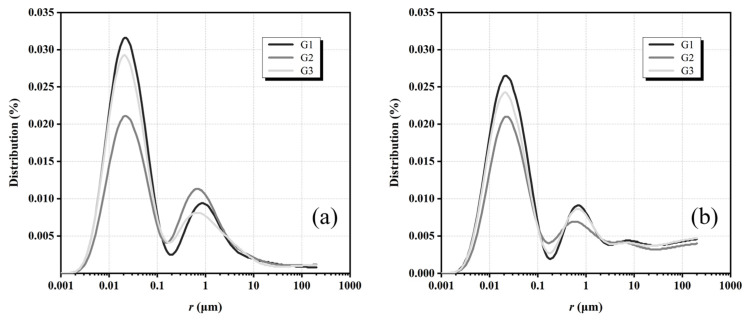
The results of low-field nuclear magnetic resonance test of the drilling core specimens for manufactured sand concrete: (**a**) standard-curing condition; (**b**) steam-curing condition.

The low-field nuclear magnetic resonance test results of manufactured sand concrete with different rock types under different curing conditions are shown in Figure 17. The calculated porosity of manufactured sand concrete per unit volume is shown in Table 6.

Figure 17 and Table 6 clearly illustrate the pore structure characteristics of manufactured sand concrete under standard-curing and steam-curing conditions. Under standard-curing conditions, the pore sizes of all manufactured sand concrete groups are mainly distributed between 0.01 and 10 μm, with relatively few large pores exceeding 10 μm. Under steam-curing conditions, the number of pores in the 0.01–10 μm range significantly decreases for all groups, while the number of large pores above 1 μm increases substantially. This indicates that the steam-curing regime increases the number of coarse pores in manufactured sand concrete, confirming the existence of pore structure coarsening in steam-cured manufactured sand concrete. Additionally, under per unit volume conditions, group G1 shows the highest porosity under both curing conditions, group G2 the lowest, and group G3 intermediate values. Under standard curing, the porosity of group G1 reaches 1.37%. Appropriately reducing the granite-manufactured sand content slightly decreases concrete porosity while maintaining values lower than limestone-manufactured sand concrete, with group G2 achieving the lowest porosity (1.15%), approximately 16.1% lower than group G1. Under steam curing, pores in the 0.01–10 μm range gradually transform into larger pores exceeding 1 μm, including harmful and multi-harmful pores, yet group G2 maintains the lowest porosity at only 1.13%.

It is explicitly stated that under steam curing, group G1 experiences microcracks in the ITZ due to a mismatch in the coefficient of thermal expansion between the aggregate and the paste, which not only increases capillary water absorption but also directly leads to a decrease in the dynamic elastic modulus and an increase in the damping ratio. These findings align with conclusions from previous low-field nuclear magnetic resonance tests. Granite-manufactured sand indeed damages the internal microstructure of concrete, and this damage is exacerbated by steam-curing technology, ultimately leading to performance degradation in granite-manufactured sand concrete. However, partial replacement of river sand or limestone-manufactured sand with appropriate amounts of granite-manufactured sand remains feasible for producing manufactured sand concrete that meets engineering requirements.

## 4. Conclusions

- Under both standard-curing and steam-curing conditions, limestone-manufactured sand concrete exhibited the highest compressive strength, splitting tensile strength, and dynamic elastic modulus, along with the lowest damping ratio, indicating fewer internal defects in specimens. After introducing granite-manufactured sand, all these parameters of concrete decreased, showing significant reductions with an increasing granite-manufactured sand content, accompanied by rapid growth in the damping ratio.
- The dry shrinkage deformation of limestone-manufactured sand concrete remained the lowest throughout the entire testing period, with limited increase under steam-curing conditions. After introducing granite-manufactured sand, the dry shrinkage deformation of concrete significantly increased throughout the testing period under both standard-curing and steam-curing conditions, while its volume stability sharply declined.
- Under both standard-curing and steam-curing conditions, the capillary absorption coefficient, electric flux, and porosity of granite-manufactured sand concrete were the highest, which were related to the inherent defects and surface characteristics of the manufactured sand raw materials as well as the application of steam-curing regimes. In comparison, the durability of limestone-manufactured sand concrete was better.
- The interfacial shear strength of granite-manufactured sand concrete was consistently lower than that of limestone-manufactured sand concrete under both standard-curing and steam-curing conditions, with a significant decrease in cohesion between the parent rock of manufactured sand and the cement paste, resulting in weaker interfacial bonding conditions.
- This conclusion provides insights for improving the fundamental properties and meso- and microstructures of manufactured sand concrete, i.e., enhancing the interfacial bonding performance between manufactured sand and cement paste (or mortar), rationally optimizing the dosage of manufactured sand and improving its surface characteristics, while adjusting and optimizing the steam-curing regime, thereby demonstrating the potential for producing high-performance manufactured sand concrete.
- This study only conducted one gradation, one dosage of cementitious material, and a specific curing system (standard curing and 60 °C steam curing). The results of granite-manufactured sand from different origins may vary. It is suggested for future research to explore the repairing effect of different steam-curing systems (such as variable-temperature curing) on granite-manufactured sand concrete.

## Figures and Tables

**Figure 1:**
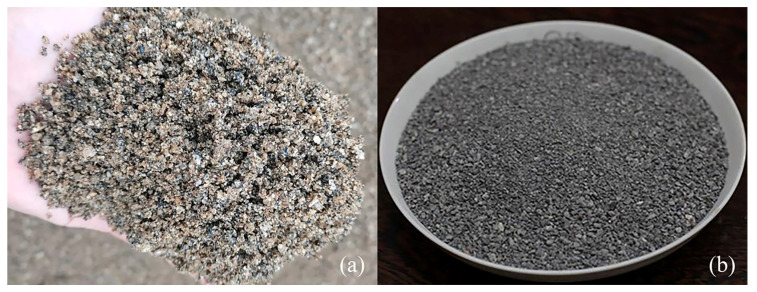
The manufactured sands used in this study: (**a**) granite-based sand; (**b**) limestone-based sand.

**Table 1:** Mechanical strength of cement.

Strength	Flexural Strength (MPa)			Compressive Strength (MPa)		
	3 d	7 d	28 d	3 d	7 d	28 d
Tested value	5.6	7.2	9.3	24.8	33.6	48.6

**Table 3:** Mix proportions of manufactured sand concretes (kg/m^3^).

Group	PC	FA	GGBS	S1	S2	G		W	SP
						5–10 mm	10–20 mm		
G1 (100% granite)	336	96	48	745	\	446	669	158.4	3.36
G2 (100% limestone)	336	96	48	\	745	446	669	158.4	3.36
G3 (50% blend)	336	96	48	372.5	372.5	446	669	158.4	3.36

**Table 4:** Fitting results of capillary adsorption coefficients of manufactured sand concrete.

Curing Scheme	Fitting Interval of *t*^0.5^	Group	Fitting Formula	*R* ^2^
Standard curing	0~10.9545	G1	*i* = 0.02319 + 0.01407·*t*^0.5^	0.9437
		G2	*i* = 0.02432 + 0.00836·*t*^0.5^	0.9534
		G3	*i* = 0.01838 + 0.01281·*t*^0.5^	0.9538
	13.4161~100.3992	G1	*i* = 0.15373 + 0.00402·*t*^0.5^	0.9798
		G2	*i* = 0.08942 + 0.00310·*t*^0.5^	0.9853
		G3	*i* = 0.14916 + 0.00337·*t*^0.5^	0.9844
Steam curing	0~10.9545	G1	*i* = 0.04249 + 0.02013·*t*^0.5^	0.9051
		G2	*i* = 0.02008 + 0.01249·*t*^0.5^	0.9465
		G3	*i* = 0.03260 + 0.01821·*t*^0.5^	0.9147
	13.4161~100.3992	G1	*i* = 0.25169 + 0.00432·*t*^0.5^	0.9429
		G2	*i* = 0.14509 + 0.00344·*t*^0.5^	0.9841
		G3	*i* = 0.20940 + 0.00309·*t*^0.5^	0.9797

**Table 5:** Shear strength parameters of manufactured sand parent rock and hardened cement paste.

Group	Cohesion (MPa)	Internal Friction Angle (°)	Fitting Formula
SB	1.76	41.40	*y* = 0.88·*x* + 1.76
SZ	1.59	41.93	*y* = 0.90·*x* + 1.59
HB	1.20	41.93	*y* = 0.90·*x* + 1.20
HZ	0.81	42.37	*y* = 0.91·*x* + 0.81

**Table 6:** Calculation results of porosity of unit volume manufactured sand concrete (%).

Curing Scheme	Calculated Porosity (%)		
	G1	G2	G3
Standard curing	1.37	1.15	1.31
Steam curing	1.35	1.13	1.28

## Data Availability

The original contributions presented in this study are included in this article. Further inquiries can be directed to the corresponding authors.
